# Cultural and religious influences on parental knowledge of HPV infection and female vaccination in Egypt: a national cross-sectional study

**DOI:** 10.1186/s12889-026-26381-w

**Published:** 2026-02-17

**Authors:** Mohamed Saad Rakab, Mohamed Baklola, Hadeer Hafez, Safaa Hassan Zaki Abbas, Mohamed Abdelrazik, Nada M. Radwan, Heba Abdelaziz Husseiny, Omnia Samy El-Sayed, Mohamed Terra, Basel Refky

**Affiliations:** 1 Faculty of Medicine, Mansoura University, Mansoura, Egypt; 2 Faculty of Medicine, October 6th University, Cairo, Egypt; 3 Alexandria Clinical Research Administration, Alexandria Health Affairs Directorate, Alexandria, Egypt; 4 Faculty of Medicine, Al-Azhar University, Nasr City, Cairo, Egypt; 5 Faculty of Medicine, Cairo University, Giza, Egypt; 6 Faculty of Medicine, Zagazig University, Zagazig, Egypt; 7 Lecturer at Oncology Centre, Mansoura University, Mansoura, Egypt

**Keywords:** HPV, Parental knowledge, Vaccination, Egypt, Cultural influences, Religious beliefs, Cervical cancer prevention

## Abstract

**Background:**

Human Papilloma Virus (HPV) is one of the most prevalent sexually transmitted infections worldwide, significantly contributing to cervical and other cancers. In Egypt, where cultural and religious norms shape health perceptions, HPV prevention, especially vaccination, remains a critical public health issue. This study investigates parental knowledge and attitudes towards HPV infection and vaccination in Egypt, focusing on the influence of cultural and religious beliefs.

**Methods:**

A national cross-sectional survey was conducted from January to April 2024 among 776 Egyptian parents. Participants were recruited using convenience and snowball sampling methods. A comprehensive questionnaire, translated and validated in Arabic, assessed knowledge about HPV and cervical cancer, beliefs about HPV vaccination, and vaccine acceptability. Data were analyzed using SPSS version 28, applying non-parametric tests to examine associations between demographic factors and study outcomes.

**Results:**

While 38.5% of parents had heard of HPV, only 26.2% knew about the HPV vaccine. Higher education levels and income were significantly associated with better knowledge of HPV transmission and its link to cervical cancer. Cultural and religious factors played a pivotal role in shaping beliefs, with older and less educated parents showing lower levels of vaccine acceptance. Mothers were more willing than fathers to vaccinate themselves and their daughters, with willingness influenced by gender, age, and income.

**Conclusion:**

Parental awareness of HPV and its vaccine remains low in Egypt, with significant cultural and religious influences on health beliefs and behaviors. Tailored public health interventions addressing these factors are needed to improve HPV vaccine uptake and reduce the burden of HPV-related diseases in Egypt.

## Background

Human Papilloma Virus (HPV) represents one of the most common sexually transmitted infections worldwide, with a significant portion of sexually active individuals being exposed to the virus at some point in their lives [1, 2]. HPV is notably associated with various malignancies, including cervical, oropharyngeal, and genital cancers, among others [1]. High-risk HPV strains, particularly HPV 16 and 18, are responsible for a substantial majority of cervical cancer cases, highlighting the critical importance of effective HPV prevention and control measures [3].

In Egypt, the prevalence and impact of HPV on women’s health have emerged as significant concerns [4]. A landmark epidemiological study in 2021 reported that 14.3% of Egyptian women tested positive for HPV DNA, underscoring a considerable public health challenge [4]. This data is particularly alarming given that virtually all cervical cancer cases (approximately 99.8%) are linked to high-risk HPV DNA sequences, with HPV 16 and 18 implicated in about 70% of invasive cervical carcinomas [5]. These findings highlight the urgent need for comprehensive strategies to address HPV infection in Egypt, encompassing awareness, screening, and vaccination efforts.

Cervical cancer ranks as the 13th most frequent cancer among women in Egypt and the 9th among women aged 15–44, with an estimated 1,320 cases diagnosed annually and 744 deaths [6, 7]. Despite the availability of HPV vaccines like Cervarix and Gardasil, Egypt lacks a national vaccination program or government funding [8]. Similarly, cervical cancer screening initiatives are minimal, with low uptake of Pap smears and no organized national program [7]. These gaps emphasize the need for robust public health strategies, including HPV vaccination and regular screenings, to combat cervical cancer’s impact.

Cultural and religious norms play a pivotal role in shaping perceptions and behaviors related to health and disease prevention in many societies, including Egypt [9, 10]. These norms can significantly influence attitudes towards sexually transmitted infections and related preventive measures, such as HPV vaccination [11]. In neighbouring Saudi Arabia, cultural reluctance to engage in HPV screening and vaccination has been identified as a key barrier to combating the virus [12]. A study by Jradi and Bawazir (2019) found that a vast majority of parents were unaware of the HPV vaccine, and a similar proportion expressed reluctance to vaccinate their daughters, highlighting the profound impact of cultural and religious beliefs on public health initiatives [12].

The challenge is further compounded by the low level of HPV-related knowledge and awareness among the general population in the region [13, 14]. Prior research has demonstrated a critical gap in understanding HPV’s transmission modes, its association with cervical cancer, and the benefits of vaccination [15]. This lack of awareness is a significant impediment to vaccination efforts, as informed consent and active participation are essential for the success of any public health intervention [15, 16].

Given these challenges, the present study seeks to address a critical knowledge gap by evaluating the awareness and attitudes towards HPV infection and vaccination among Egyptian parents. This study is especially timely and relevant, considering the high HPV prevalence in Egypt and the potential for cultural and religious norms to influence health-seeking behaviors [17]. By shedding light on the current state of HPV knowledge and vaccine acceptance, the study aims to inform targeted educational and intervention strategies to enhance HPV awareness, promote vaccination, and ultimately reduce the burden of HPV-related diseases in Egypt.

## Methods

### Study design and population

A national cross-sectional survey was conducted in Egypt between January and April 2024 to assess the knowledge of parents regarding HPV infection and vaccination. The study targeted parents residing in various regions of Egypt, aiming for a diverse and representative sample. Participants were included based on predefined criteria, and exclusions were made for individuals under 18 years old, non-residents of Egypt, parents of male children, or those who either refused to participate or failed to complete the survey. By encompassing a broad geographic and demographic scope, the study provides a comprehensive assessment of parental knowledge across the country.

### Sample size calculation

A single proportion of the population formula: n = [(Za/2)2P(1-P)]/d2 was applied to detect the minimal sample size needed for the study. With a 95% confidence level (Z a/2 = 1.96), a 5% margin of error, P = the estimated overall test score among parents (32.9%) which was estimated from results of the pilot study, and adding 5% for a possible non-response rate, 340 individuals were needed to proceed with this study. However, to account for potential clustering effects, we applied a design effect of 2, increasing the minimum required sample size to 680. After adjusting for a possible non-response rate, the survey was completed by 776 participants.

### Sampling and data collection approach

A combination of convenience and snowball sampling methods was employed to recruit participants for this study. Data were collected using a Google Form questionnaire, which was distributed both in-person and via various social media platforms. The questionnaire clearly outlined the title, aim, and objectives of the research, ensuring participants were well-informed about the study’s purpose. Participation was entirely voluntary, and a statement was included clarifying that the completion of the questionnaire would be considered as informed consent. The survey was conducted solely for research purposes, with no obligation for respondents to participate.

### Data collection tool

The questionnaire used in this study was adapted from a previously validated questionnaire developed in Thailand, which was designed to assess parents’ knowledge, beliefs, and acceptance of HPV vaccination in relation to their socio-demographics and religious beliefs [18]. In our adaptation, the questionnaire was tailored to the Egyptian context by modifying the phrasing of some questions to reflect cultural and religious nuances. Additionally, the questionnaire was translated into Arabic to facilitate data collection among participants.

The final adapted questionnaire consisted of seven sections, each addressing different aspects relevant to the study. The section on Demographic and Professional Information contained 10 items assessing variables such as age, gender, education level, and occupation. Health-Related Lifestyle was covered with five items that explored participants’ general health practices and behaviors. The section on Background Knowledge of HPV and the HPV Vaccine included seven items that measured participants’ basic understanding of these topics. Knowledge About HPV and Cervical Cancer was addressed through 13 items that investigated participants’ awareness of the virus, its transmission, and its link to cervical cancer. Beliefs About HPV and the HPV Vaccine were assessed with 16 items that gauged participants’ attitudes toward the severity of HPV and the perceived benefits and risks of vaccination.

Finally, the section on Acceptability of HPV Vaccination included nine items that assessed participants’ willingness to vaccinate themselves or their children. The questionnaire featured a variety of question types, including Yes/No, multiple-choice, and Likert scale-based items.

### Translation and validity of the questionnaire

The questionnaire was translated from English into Arabic using a standardized forward–backward translation procedure to ensure linguistic and cultural accuracy. Two bilingual experts independently translated the English version into Arabic, and any discrepancies were resolved through discussion to produce a single reconciled version. This version was then back-translated into English by two independent translators who were blinded to the original text to verify conceptual equivalence.

To ensure content validity, a panel of eight experts in public health and microbiology evaluated the Arabic version for clarity, relevance, and cultural suitability. Experts were encouraged to suggest modifications for any items deemed unclear or inappropriate. The Content Validity Index (CVI) was determined at three levels: item (I-CVI), expert (E-CVI), and scale (S-CVI). The I-CVI was calculated by dividing the number of experts rating an item as clear and relevant (rating ≥ 3) by the total number of experts [19]. The E-CVI represented the proportion of items each expert rated as relevant, while the S-CVI was the average of all I-CVI values across items. Items with an I-CVI > 0.79 were considered appropriate, those between 0.70 and 0.79 were revised, and those below 0.70 were removed. The final S-CVI was 0.91, indicating strong content validity of the Arabic version.

### Pilot study

A pilot study was conducted among 30 Egyptian parents to evaluate the clarity, comprehensibility, and cultural appropriateness of the questionnaire. Minor modifications were made based on participant feedback to improve readability. The internal consistency of the final version was confirmed using Cronbach’s alpha, which yielded a value of 0.827, indicating good reliability.

### Statistical analysis

Once we finished collecting data, we saved it in a protected Excel file and transferred it to SPSS for analysis. We used IBM’s SPSS software, version 28, on a Windows system to enter and analyze the data. We checked the data distribution using the Shapiro-Wilk test, which showed an abnormal distribution. As a result, we used medians and interquartile ranges to describe continuous data, and for categorical data, we used descriptive statistics, frequencies, and proportions. To compare scores on different scales, we used the Mann-Whitney and Kruskal Wallis tests. If the two-sided P value was less than 0.05, we considered it statistically significant.

## Results

### Characteristics of the participants

We surveyed 776 parents of at least one daughter (Table 1). Mothers comprised the majority representing 70.2% and fathers 29.8% of the respondents. Age distribution showed that 60.7% were over 35 years old, and only 10.3% were aged 26–30. The vast majority, 93.9%, identified as Muslim. Most participants (70.6%) had a university education. Occupation varied, with 41.1% employed and 19.3% unemployed. On average, participants had 2 daughters, and 80.4% reported complete childhood vaccinations for their children. For religious importance, 54.3% considered it rather or very important. Health checks were irregular, with 40.5% never having one. Familiarity with HPV was moderate, with 38.5% having heard about it, and 26.2% knew about the HPV vaccine.

**Table 1:** Demographic and characteristics of the participants

Characteristics	Overall (*N* = 776)	Characteristics	Overall (*N* = 776)
Parent’s Gender		Childhood Vaccination	
Father	231 (29.8%)	No	26 (3.4%)
Mother	545 (70.2%)	Unsure	61 (7.9%)
Age		Yes, all	624 (80.4%)
Less than 26	105 (13.5%)	Yes, some	65 (8.4%)
26–30	80 (10.3%)	Religious Importance	
31–35	120 (15.5%)	Irreligious	4 (0.5%)
More than 35	471 (60.7%)	Neither important nor unimportant	323 (41.6%)
Religion		Not important	28 (3.6%)
Christian	47 (6.1%)	Rather important	273 (35.2%)
Muslim	729 (93.9%)	Very important	148 (19.1%)
Education Level		Health Check	
Below university education	228 (29.4%)	2–5 years interval	111 (14.3%)
University education	548 (70.6%)	More than 1–2 years interval	255 (32.9%)
Income		More than 5 years interval	96 (12.4%)
1000–5000 EGP	264 (34.0%)	Never	314 (40.5%)
5001 − 1000 EGP	215 (27.7%)	Abnormal Pap Smear (Yes)	15 (1.9%)
Less than 1000 EGP	123 (15.9%)	History of Cervical Cancer (Yes)	39 (5.0%)
More than 10,000	174 (22.4%)	Alcohol Consumption (Yes)	25 (3.2%)
Number of Daughters		Smoking (Yes)	132 (17.0%)
Mean (SD)	2.00 (1.08)	Heard about HPV (Yes)	299 (38.5%)
Median [Min, Max]	2.00 [1.00, 11.0]	Asked for HPV Information (Yes)	182 (23.5%)
Daughters Aged 9–45		Informed about HPV by Physician (Yes)	144 (18.6%)
Mean (SD)	1.64 (1.04)	Heard about HPV Vaccine (Yes)	203 (26.2%)
Median [Min, Max]	1.00 [0, 7.00]	Informed about HPV by Physician (Yes)	113 (14.6%)
		Childhood Vaccination	

### Knowledge about HPV and cervical cancer

Table 2 demonstrates all questions and associations regarding knowledge. Nearly half of the respondents (49.4%) correctly identified that HPV infection is contracted through sexual contact, while 56.4% were aware that HPV can be transmitted even without symptoms. The majority (78.4%) understood that engaging in sex at an early age increases the risk of HPV infection, and 65.1% recognized that having multiple sexual partners also increases this risk. However, only 29.4% knew that genital warts are caused by HPV, and only 33.1% were aware that most people with genital HPV show no visible symptoms. When considering demographic factors, income, religion, and education level consistently influenced knowledge levels. For example, income level was significantly associated with awareness of HPV transmission without symptoms (*p* = 0.073) and the risk increase from multiple sexual partners (*p* = 0.021). Educational level was particularly influential, showing strong associations with overall knowledge (*p* = 0.035) and awareness of HPV’s link to cervical cancer (*p* < 0.001). Interestingly, gender did not significantly affect overall knowledge, though it was linked to specific aspects, such as the ability to identify the transmission of HPV without symptoms (*p* = 0.006).

**Table 2:** Knowledge about HPV and cervical cancer

	Correct answer, *n*. (%)	Gender	Age	Income	Religion	Level of education
HPV infection is contracted by sexual contact.	383 (49.4)	0.885	0.544	0.006*	0.012*	< 0.001*
People can transmit HPV to their partner(s) even if they have no symptoms of HPV infection.	438 (56.4)	0.006*	0.742	0.073	0.210	0.0.229
Having multiple sexual partners’ increases risk of HPV infection.	505 (65.1)	0.642	0.238	0.021*	0.029*	0.448
Sex at early age increases risk of HPV infection.	608 (78.4)	0.302	< 0.001*	0.300	0.279	0.030*
Genital warts are caused by HPV infection.	228 (29.4)	0.071	0.058	0.004*	0.504	0.007*
Most people with genital HPV have no visible signs or symptoms.	257 (33.1)	0.023	0.524	0.223	0.207	0.051
HPV infection can be prevented by vaginal douching after intercourse.	101 (13.0)	< 0.001*	0.047*	0.100	0.055	0.022*
HPV infection can be treated by antibiotics.	43 (5.5)	0.014*	0.127	0.008*	0.011*	0.062
Smoking increases risk of cervical cancer.	376 (48.5)	0.126	0.084	0.048*	0.004*	0.951
HPV infection can cause cervical cancer.	448 (57.7)	0.623	0.018*	< 0.001*	< 0.001*	< 0.001*
Cervical cancer symptoms commonly present with vaginal discharge or bleeding even in the early stages of disease.	153 (19.7)	0.016*	0.011*	0.041*	0.024*	0.013*
A Pap smear is only indicated in women with vaginal discharge or bleeding.	89 (11.5)	0.489	0.769	0.211	0.421	0.581
Unmarried women are not supposed to have a Pap smear.	125 (16.1)	0.346	0.004*	0.014*	0.054	< 0.001*
Overall knowledge, median (IQR)	3.85 (2.31–5.38)	0.271	0.504	< 0.001*	0.004*	0.035*
* indicates statistical significance at the 5%level (p < 0.05)						

### Beliefs about HPV and HPV vaccine

Table 3 outlines respondents’ beliefs about HPV and the HPV vaccine, with agreement levels on a 5-point scale. A significant proportion of participants agreed that HPV infection is a serious health concern, with a mean agreement score of 3.74 (SD = 0.889), and cervical cancer is also recognized as a serious disease (mean = 3.48, SD = 0.878).

**Table 3:** Beliefs about HPV and HPV vaccine

	Agreement, mean (SD)	Gender	Age	Income	Religion	Level of education
There is a risk for young women to contract HPV	3.60 (0.837)	0.398	0.038*	0.004*	0.102	0.346
There is a risk for young women to contract cervical cancer	3.51 (0.835)	0.279	0.247	< 0.001*	0.003*	0.364
HPV infection is a serious health concern	3.74 (0.889)	0.183	0.837	0.059	0.453	0.025*
Cervical cancer is a serious disease	3.48 (0.878)	0.461	0.959	0.001*	0.944	0.008*
The HPV vaccine is effective in preventing condyloma	3.38 (0.802)	0.266	0.426	0.171	1.000	0.064
The HPV vaccine is effective in preventing cervical cancer	3.30 (0.805)	0.024*	0.376	0.095	0.735	0.421
I have trust in the HPV vaccination	3.14 (0.859)	0.490	0.150	0.224	0.603	0.060
The HPV vaccine can cause adverse effects	2.99 (0.908)	0.002*	0.190	0.002*	0.038*	0.326
It is problematic that HPV vaccination requires three injections	3.69 (0.929)	0.324	0.555	0.017*	0.031*	0.831
The efficiency of HPV vaccine is unclear	3.65 (0.891)	0.349	0.606	0.059	0.085	0.004*
The HPV vaccine is harmful	3.42 (0.814)	0.614	0.698	0.199	0.864	0.129
Women who have been HPV vaccinated should have Pap smear annually	3.16 (0.954)	0.008*	0.802	0.024*	0.496	0.936
HPV vaccination decreases condom use	3.37 (1.122)	0.560	0.811	0.002*	0.016*	< 0.001*
HPV vaccination causes my daughter to be sexually active early	3.29 (1.071)	0.312	0.435	0.423	0.147	0.208
HPV vaccination increases number of sexual partners	3.26 (0.895)	0.766	0.978	0.991	0.958	0.680
HPV vaccination increases awareness of sexually transmitted diseases	3.57 (0.887)	0.615	0.041*	0.313	0.814	0.849
Overall beliefs, mean (SD)	6.82 (0.873)	0.344	0.435	0.057	0.044*	0.104
* indicates statistical significance at the 5%level (p < 0.05)						

Age and income showed strong associations with several beliefs. For example, older age groups were less likely to recognize the risk of young women contracting HPV (*p* = 0.038) and cervical cancer (*p* = 0.247), while higher income was linked to greater concern about HPV infection (*p* = 0.004) and cervical cancer (*p* < 0.001). Educational level also influenced beliefs, with those of higher income more likely to perceive HPV infection as a serious health concern (*p* = 0.025) yet believed that the efficiency of HPV vaccine is still unclear (*p* = 0.004). Gender was significantly associated with the belief that the HPV vaccine is effective in preventing cervical cancer (*p* = 0.024). However, there was no significant gender difference in trust in the HPV vaccination or in beliefs about its safety.

### Acceptability of HPV vaccination

Respondents showed a general willingness to support HPV vaccination initiatives, with a mean agreement score of 3.69 (SD = 1.163) for offering the vaccine free to 9–11-year-old girls, though this was not significantly influenced by demographic factors (Table 4). Support for vaccinating every child had a mean score of 3.41 (SD = 1.180) and varied significantly by age (*p* < 0.001) and income (*p* = 0.021). Mothers’ willingness to vaccinate themselves had a mean score of 3.41 (SD = 1.117), significantly associated with gender (*p* = 0.036), age (*p* < 0.001), and income (*p* = 0.011). Acceptance of HPV vaccination for their daughters had a mean score of 3.43 (SD = 1.164), with significant associations to gender (*p* = 0.044) and age (*p* = 0.002). Willingness to pay for the vaccine if outside the target group had a mean score of 3.06 (SD = 1.182), significantly influenced by age (*p* < 0.001) and education (*p* < 0.001). The overall acceptability score of 6.97 (SD = 1.652) was significantly related to age (*p* < 0.001) and income (*p* = 0.029).

**Table 4:** Acceptability of HPV vaccination

	Agreement, mean (SD)	Gender	Age	Income	Religion	Level of education
Do you agree if Ministry of Public Health is going to offer HPV vaccine for free to 9–11 years old girls?	3.69 (1.163)	0.815	0.413	0.099	0.901	0.331
Do you agree with the policy of giving vaccination to every child?	3.41 (1.180)	0.244	< 0.001*	0.021*	0.555	0.978
Do you consider vaccinating yourself (for mother)?	3.41 (1.117)	0.036*	< 0.001*	0.011*	0.225	0.028*
Will you accept active HPV vaccination of your daughter?	3.43 (1.164)	0.044*	0.002*	0.597	0.565	0.597
If your daughter is not in the target group, do you still want to pay for vaccinating her?	3.06 (1.182)	0.067	< 0.001*	0.210	0.332	< 0.001*
If the government offers HPV vaccination for free, I will vaccinate my daughter	3.44 (1.145)	0.014*	< 0.001*	0.164	0.531	0.177
Now HPV vaccine costs 6000–6900 baht per course, yet I will vaccinate my daughter	3.15 (1.152)	0.267	< 0.001*	0.454	0.208	0.016*
I don’t have enough information about HPV vaccine to decide whether to give it to my daughter	3.92 (1.176)	0.979	0.149	0.003*	0.076	< 0.001*
HPV vaccine is so new that I want to wait a while before deciding if my daughter should get it	3.85 (1.151)	0.227	0.452	< 0.001*	0.203	< 0.001*
Overall acceptability, mean (SD)	6.97 (1.652)	0.052	< 0.001*	0.029*	0.745	0.708
* indicates statistical significance at the 5%level (p < 0.05)						

## Discussion

Human papillomavirus (HPV) infection stands as one of the most prevalent sexually transmitted infections [20]. HPV genotypes are classified as either low-risk or high-risk. Infection with low-risk HPV types (HPV-6 or HPV-11) can lead to the development of genital warts, but they typically do not progress to cancer [21]. Conversely, infection with high-risk HPV types such as HPV-16 and 18 is frequently associated with various malignancies, including vaginal, cervical, vulvar, penile, or anal cancers [22].

Notably, HPV-16 and HPV-18 have been identified as the causative agents in approximately 70% of cervical cancer cases [23]. In alignment with the World Health Organization’s 2020 global strategy, there is an urgent need to accelerate efforts towards the elimination of cervical cancer by 2030. This ambitious goal is underpinned by three key targets: ensuring 90% of girls are vaccinated against HPV by age 15, facilitating 70% of women to undergo high-performance testing (comparable to or surpassing the HPV test) by ages 35 and 45, and guaranteeing that 90% of women diagnosed with cervical disease receive appropriate treatment [24]. These measures collectively aim to mitigate the burden on healthcare systems and reduce cervical cancer-related mortality rates. In light of these objectives, the present study sought to assess Egyptian parents’ knowledge about cervical cancer, HPV, and the HPV vaccine, given the critical role parental awareness plays in the decision to vaccinate their daughters against HPV.

Our findings reveal that awareness and knowledge levels regarding HPV and its vaccine among Egyptian parents are relatively low compared to other studies. Only 38.5% of parents in our sample had heard of HPV, with an even smaller proportion (26.2%) aware of the HPV vaccine. These results align with findings from other countries where HPV awareness is generally low, particularly in regions where HPV vaccination has not been widely promoted or integrated into public health programs [25, 26].

For instance, studies conducted in Saudi Arabia and Thailand have reported similarly low awareness levels, with only a minority of participants demonstrating adequate knowledge about HPV and its connection to cervical cancer [27, 28]. The relatively low levels of awareness observed in our study could be attributed to similar cultural and educational factors seen in these countries, where discussions surrounding sexually transmitted infections (STIs) are often limited due to social and religious sensitivities [29, 30].

Interestingly, our data indicate that certain socio-demographic factors significantly impact HPV knowledge. Education level, for example, is strongly associated with knowledge about HPV transmission and prevention. This finding aligns with international studies, which often cite higher education as a key determinant of better understanding of HPV-related health risks [31]. Furthermore, income level plays a crucial role, with parents of higher socioeconomic status demonstrating significantly better knowledge about HPV. This trend mirrors observations in other developing nations where socioeconomic status influences access to health information [32].

Religious beliefs also emerged as a significant factor influencing knowledge, with Muslim participants being less likely to have accurate information about HPV compared to their Christian counterparts. This finding could be linked to differences in approaches to sexual health education within various religious contexts, a trend that has been observed in other predominantly Muslim countries [33–35].

The findings reveal significant gaps in knowledge about HPV and its vaccine among Egyptian parents. Despite the fact that 57.7% of participants correctly identified that HPV infection can cause cervical cancer, only 5.5% were aware that HPV cannot be treated with antibiotics, and 13.0% mistakenly believed that vaginal douching could prevent HPV infection. This highlights a critical misunderstanding of HPV prevention and treatment, which could undermine efforts to increase vaccination uptake [36].

Moreover, there is a considerable knowledge gap regarding the purpose and necessity of Pap smears, with only 16.1% of participants disagreeing with the misconception that unmarried women should not have a Pap smear. This is concerning, as it reflects a lack of awareness about the importance of cervical cancer screening, a gap that has been similarly identified in other low-awareness contexts [37, 38].

The low levels of knowledge about the HPV vaccine’s efficacy and safety are also notable. While the mean agreement score on the statement that the HPV vaccine is harmful was 3.42 (SD 0.814), the overall belief score was 6.82 (SD 0.873), indicating moderate skepticism. This suggests that even among those who are aware of the vaccine, there is hesitation and uncertainty, which could be due to a lack of comprehensive health education. This hesitation mirrors findings from other countries where the introduction of the HPV vaccine has faced public skepticism, often due to misinformation or insufficient public health campaigns [39].

These gaps in knowledge underscore the need for targeted educational interventions in Egypt. Public health campaigns should focus on clarifying misconceptions about HPV transmission, prevention, and the purpose of the HPV vaccine. By addressing these gaps, it may be possible to increase the overall acceptability of the vaccine, as the data suggests that willingness to vaccinate daughters increases significantly when parents are better informed [40].

Religion and cultural factors significantly influence the perceptions and acceptability of the HPV vaccine among Egyptian parents. The study revealed that Muslim parents were generally less knowledgeable about HPV and its vaccine compared to Christian parents (*p* < 0.001). This could be due to cultural sensitivities around discussing sexually transmitted infections (STIs) in predominantly Muslim societies, where conversations about sexual health are often taboo. Such cultural barriers may lead to lower levels of public health education and awareness, contributing to misinformation and reluctance to engage with preventative healthcare measures like the HPV vaccine [41, 42].

Furthermore, religious importance was a strong determinant in shaping parents’ attitudes toward HPV vaccination. Those who considered religion to be very important were more likely to hold misconceptions about the vaccine, such as believing it could encourage early sexual activity or increase the number of sexual partners. This mirrors findings from other studies in similar cultural contexts, where religious beliefs sometimes conflict with modern medical practices, leading to lower vaccine acceptance rates [43].

Cultural norms also play a role in shaping these perceptions. In many traditional Egyptian communities, there is a strong emphasis on modesty and the protection of female chastity, which can influence attitudes toward a vaccine associated with a sexually transmitted infection. Parents may fear that vaccinating their daughters could be perceived as endorsing or anticipating sexual activity, leading to hesitancy or outright refusal of the vaccine [44–46].

The current state of cervical cancer prevention in Egypt further underscores the urgency of addressing the gaps identified in this study. Despite the availability of highly effective HPV vaccines, such as Cervarix and Gardasil, there is no national vaccination program or government funding for HPV vaccination in Egypt [7]. Similarly, cervical cancer screening initiatives, including Pap smear testing, remain sparse, with low levels of uptake among eligible women [6]. This lack of structured national programs likely exacerbates the low awareness and skepticism surrounding HPV and its vaccine observed in our findings. Establishing government-supported vaccination and screening programs, alongside culturally tailored public health campaigns, is crucial to improving HPV prevention efforts in Egypt.

In line with the World Health Organization’s 90-70-90 strategy for cervical cancer elimination [47], which aims for 90% of girls to be fully vaccinated against HPV by age 15, 70% of women to be screened with a high-performance HPV test by ages 35 and 45, and 90% of women with cervical disease to receive appropriate treatment, Egypt would greatly benefit from adopting molecular HPV-based screening methods [48]. Molecular testing, particularly HPV DNA testing, has been shown to provide higher sensitivity and longer-term protection compared to conventional cytology for the detection of high-grade cervical lesions. The introduction of self-sampling for HPV testing has also been demonstrated in several studies to increase screening participation, particularly among women who face cultural, religious, or logistical barriers to clinic-based screening [49]. Integrating these evidence-based strategies with vaccination programs and culturally tailored public health interventions could substantially strengthen national efforts to prevent cervical cancer and support Egypt’s alignment with global elimination targets.

### Limitations

This study has several limitations that should be acknowledged. First, the use of a convenience sampling method poses a key limitation, potentially introducing selection bias and limiting the generalizability of the findings. The small sample size and underrepresentation of parents from rural areas further constrain the study’s ability to reflect the diverse perspectives and access to healthcare information across Egypt. Additionally, the exclusion of parents of male individuals prevents insights into attitudes toward gender-neutral HPV vaccination programs, which are being adopted in many countries. This exclusion may overlook potential differences in vaccine acceptability influenced by cultural and religious factors. Finally, the reliance on self-reported data introduces the possibility of bias due to social desirability or recall inaccuracies. These limitations highlight the need for further research with more representative sampling and inclusion of diverse parental groups.

## Conclusion

This study provides valuable insights into participants’ knowledge, beliefs, and acceptability of HPV vaccination. The findings highlight a moderate level of awareness regarding HPV and its association with cervical cancer, though gaps in knowledge persist, particularly in understanding the vaccine’s benefits. Beliefs surrounding the safety and efficacy of HPV vaccination influence acceptance rates, underscoring the need for targeted educational interventions. Enhancing public knowledge and addressing misconceptions could improve vaccine uptake and support public health initiatives aimed at reducing HPV-related diseases. Future research should explore the impact of tailored awareness campaigns and examine long-term vaccine acceptance trends.

## Data Availability

The datasets used during the current study are available from the corresponding author upon reasonable request.
